# Circular RNAs: Promising Targets in Osteoporosis

**DOI:** 10.1007/s11914-023-00786-7

**Published:** 2023-04-29

**Authors:** Sara Reis Moura, Maria João Fernandes, Susana G. Santos, Maria Inês Almeida

**Affiliations:** 1 i3S – Instituto de Investigação e Inovação em Saúde, Universidade do Porto, Porto, Portugal; 2 INEB – Instituto de Engenharia Biomédica, Universidade do Porto, Porto, Portugal; 3 ICBAS – Instituto de Ciências Biomédicas Abel Salazar, Universidade do Porto, Porto, Portugal

**Keywords:** Transcriptome, Osteogenic differentiation, Osteoclastogenesis, Bone

## Abstract

**Purpose of Review:**

Circular RNAs (circRNAs) are RNA transcripts derived from fragments of pre-messenger RNAs through a back-splicing process. An advantage that rises from their circular covalently closed conformation is their high stability, when compared with their linear counterparts. The current review focuses on the emerging roles of circRNAs in osteoporosis, including in osteogenic differentiation and osteoclastogenesis. Their potential as osteoporosis biomarkers will also be discussed.

**Recent Findings:**

Although firstly described as non-coding, some of these single-stranded RNAs were recently reported to possess protein-coding capacity. On the other hand, the circRNAs exhibit cell and tissue-specific patterns at the transcriptome level in eukaryotes and are regulated throughout the development or disease progression. Even though thousands of these circular transcripts are listed and annotated, only a limited number of studies describe their biological role in bone processes. Recent evidence indicates inhibitory activator roles in both osteoblasts and osteoclasts differentiation and function. Latest screenings in the blood, plasma, or serum of osteoporosis patients support the potential for circRNA signature to be used as biomarkers in osteoporosis, but further validation is required.

**Summary:**

While intense research into circRNAs has been detailing their biological roles, there remains a need for standardization and further research to fulfil the future potential of this emerging and highly promising class of regulatory molecules.

## Introduction

Circular RNAs (circRNAs) are single-stranded and covalently closed RNA molecules generated from the linear pre-messenger RNAs (mRNAs) via a back-splicing mechanism, in which a downstream 5′ splice site is linked to an upstream 3′ splice site, producing a closed continuous conformation [1]. This new class of RNA transcripts is present in eukaryotic cells and has been increasingly investigated in the most recent years [2, 3]. Circularized RNA molecules were initially identified in 1976 in plant viroids [4] and were later observed by electron microscopy in the mammalian HeLa cell line [5]. In 1991, the Vogelstein lab described RNA products from the tumor suppressor DCC gene, in which the first nucleotide of an originally upstream exon was linked to the last nucleotide of an originally downstream exon [6]. Surprisingly, the exons joined in a different order from that present in the genome [6]. Later, in 1993, the Lovell-Badge lab reported the presence of circular RNA molecules from the Sry locus gene in mouse testis [7]. Interestingly, this circular Sry transcript was present in the cytoplasm rather than the nucleus [7]. Most recently, the association of abnormal circRNA levels with multiple diseases has triggered a deeper analysis and extensive research on this new class of molecules.

The large amount of data generated by high-throughput sequencing methods allowed the identification of circRNA unique disease signatures, with oncology being the propellent driver for the expansion into other medical fields, including orthopedics and regenerative medicine. Only recently, the first studies profiling circRNA in osteoporotic patients were published [8]. However, while the expression levels of circRNA in bone cells have been extensively investigated, assessing the functional roles of circRNA remains a challenge and requires attention. Whether circRNAs are by-products of aberrant splicing or active functional players in cellular mechanisms remains an open question. An important technical limitation to consider for functional studies using small interfering RNAs (siRNAs) against circRNAs is that the siRNA molecules should be designed to target a specific circRNA, without affecting the linear transcripts.

Increasing evidence shows that circRNA can function as microRNA (miRNAs) sponges, working as competing endogenous RNAs (ceRNAs) [9, 10•]. In 2013, the research teams of Kjems [11] and Rajewsky [12] independently described circRNAs as functional sponges. Specifically, the Kjems lab reported that the circular transcript ciRS-7 (circular RNA sponge for miR-7) could suppress miR-7 activity while upregulating the levels of miR-7 targets [11]. Also, the authors showed that the testis-specific circRNA Sry serves as a sponge for miR-138 [11]. On the other hand, the Rajewsky lab proved that the circRNA CDR1 antisense (CDR1as) harbors conserved binding sites for miR-7 and it functions as a negative regulator of miR-7 [12]. Additionally, circRNAs can play a role as modulators of transcription and translation [10•].

Although circRNAs are usually categorized as non-coding, it was recently shown that these molecules can be translated into proteins [13]. This is the case of circ-ZNF609 that regulates myoblast proliferation, and which contains an open reading frame encoding a protein in a splicing-dependent and cap-independent manner [14]. Also, Pamudurti et al. demonstrated that a subset of circRNAs could be translated and associates with translating ribosomes in vivo [15].

circRNAs are also relevant disease biomarkers and have been detected in the blood and in the bone tissue of osteoporosis patients. This class of transcripts is more stable than their linear RNA counterparts, being more resistant to exonuclease digestion, partially due to the lack of a 5′ cap and a 3′ tail [16]. This represents an advantage for the detection of circRNA circulating levels. Moreover, circRNAs have been shown to be present in extracellular vesicles, including microvesicles and exosomes [17]. Importantly, circRNAs are enriched in extracellular vesicle preparations over their linear counterparts [18]. However, methodologies for extracellular vesicle isolation and exosomal circRNA detection should be improved and standardized to allow comparison between studies.

**Fig. 1:**
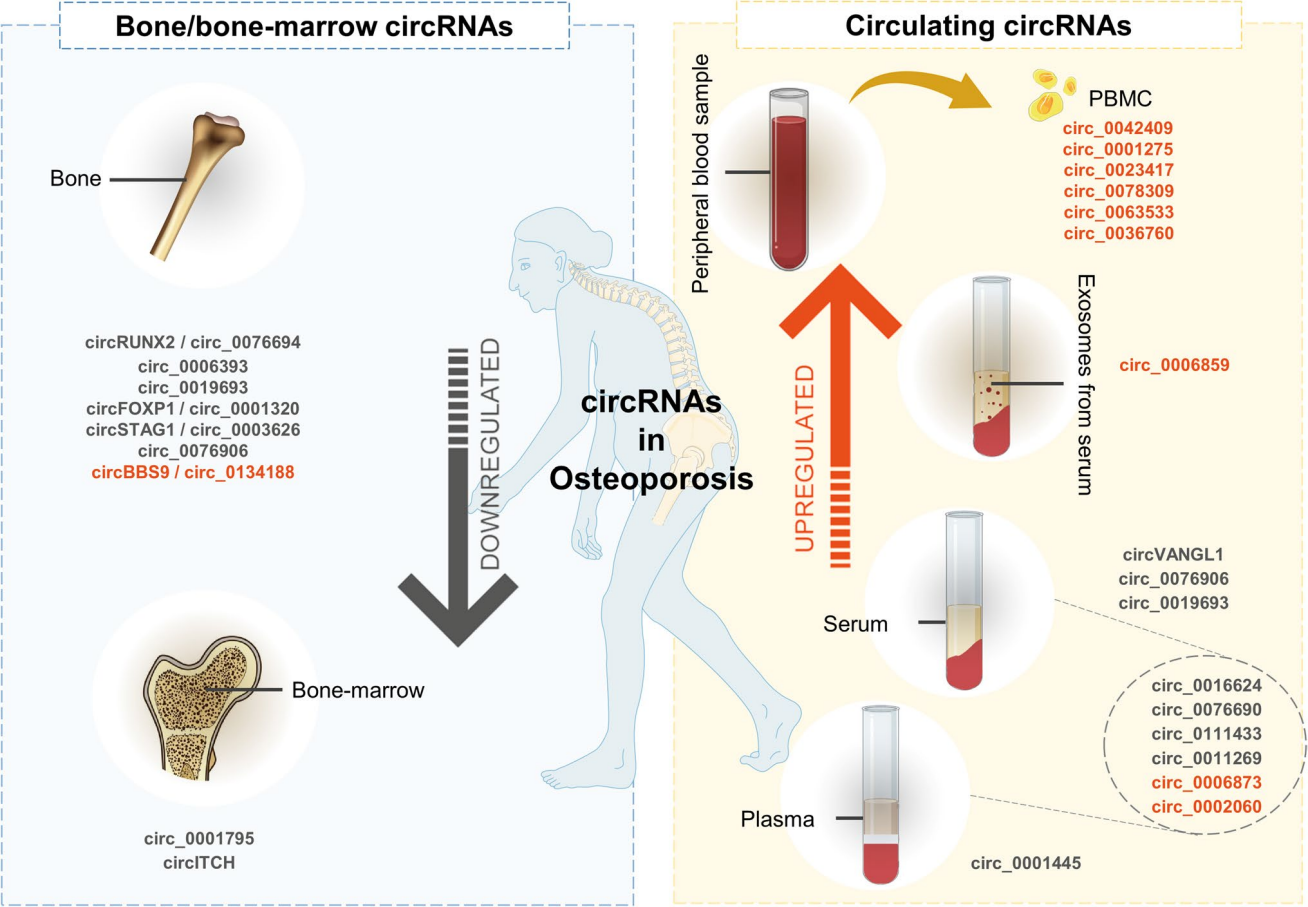
Circular RNAs (circRNAs) in the bone/bone-marrow (left panel) and circulating circRNAs (right panel) in human samples from osteoporotic patients. Red, upregulated; grey, downregulated

Currently, thousands of circRNAs have been annotated in databases, including circBase, circBank, CIRCpedia, circAtlas, circNET, Circ2Traits, and exoRbase, the majority of which with yet unknown functions [19, 20]. This review focuses on studies that explore the functional role of circRNA in bone cells and in bone-related mechanisms, including osteogenic differentiation and osteoclastogenesis. Furthermore, studies that explore circulating circRNA as biomarkers of osteoporosis in human patients are also covered (Fig. 1). As no standards for circRNA nomenclature are widely applied [20], herein the nomenclature used in the original articles will be maintained.

## Role of circRNAs in Osteogenic Differentiation

In osteoporosis, the bone remodeling process is disrupted due to an enhanced activity of osteoclasts, which is not compensated by an increase in bone formation by the osteoblasts [21, 22]. Therefore, there is the need to investigate therapeutic approaches that could modulate the differentiation and function of osteoblasts [23, 24]. Several studies used high-throughput technologies, including RNA-sequencing and microarrays, to identify circRNA signatures during osteogenic differentiation [25–29]. Although these methodologies are needed for screening profiling, it is important to refine the study of the circRNA candidates and to explore their individual role in osteogenic differentiation and on bone formation-related processes. In this section, we will mainly focus on studies that investigate the functional role of circRNAs in osteogenic differentiation in human primary cells. Table 1 summarizes the circRNA involved in the inhibition or enhancement of osteogenic differentiation in in vitro studies.

**Table 1:** Circular RNAs (circRNAs) involved in osteogenic differentiation in in vitro studies

circRNA	Type of cells	Species	Expression during osteogenic differentiation (**↑**/**↓**)	Biological process affected	Function/phenotype	In vivo	Ref
circ_0003865	BMSC*^1^	Human	**↓**	Differentiation/mineralization	Anti-osteogenic and anti-mineralization	x	[31]
circIGSF11/hsa-circRNA13685	BMSC	Human	**↓**	Differentiation/mineralization	Anti-osteogenic and anti-mineralization		[27]
circMCM3AP	ASC*^2^	Human	**↓**	Differentiation/mineralization	Anti-osteogenic and anti-mineralization		[28]
circPOMT1	ASC	Human	**↓**	Differentiation/mineralization	Anti-osteogenic and anti-mineralization		[28]
circUSP45/hsa_circ_0077425	BMSC	Human		Differentiation/mineralization/proliferation	Anti-osteogenic, anti-mineralization and anti-proliferation	x	[36]
circCDK8/hsa_circ_0003489	PDLSC*^3^	Human		Differentiation/mineralization/apoptosis/autophagy	Anti-osteogenic, anti-mineralization, pro-autophagy, and pro-apoptosis		[37]
hsa_circ_0006859	BMSC	Human	**↓**	Differentiation/mineralization	Anti-osteogenic, pro-adipogenic and anti-mineralization		[34••]
circHGF/hsa_circ_0080914	BMSC	Human		Differentiation/mineralization/proliferation	Anti-osteogenic, anti-mineralization and anti-proliferation		[35]
circRNA CDR1as/hsa_circ_0001946	BMSC^a^	Human		Differentiation/mineralization	Anti-osteogenic, pro-adipogenic and anti-mineralization		[40]
circ_0058792	MC3T3	Mice	**↓**	Differentiation	Anti-osteogenic		[38]
circRNA CDR1as/hsa_circ_0001946	PDLSC	Human	**↑**	Differentiation/mineralization	Pro-osteogenic and pro-mineralization	x	[39]
circFOXP1/hsa_circ_0001320	ASC	Human	**↑**	Differentiation/mineralization	Pro-osteogenic and pro-mineralization	x	[48]
circ_0001795	BMSC	Human	**↑**	Differentiation/mineralization	Pro-osteogenic and pro-mineralization		[49]
circ_0005564	BMSC	Human	**↑**	Differentiation/mineralization	Pro-osteogenic and pro-mineralization		[25]
hsa_circ_0006215	BMSC	Human	**↑**	Differentiation/mineralization (supernatant affect angiogenesis)	Pro-osteogenic and pro-mineralization (pro-invasion, pro-migration and pro-angiogenesis—HUVECS)	x	[47••]
circ_0011269	BMSC	Human	**↑**	Differentiation	Pro-osteogenic		[50]
circ_0019693	BMSC	Human	**↑**	Differentiation (supernatant affect angiogenesis)	Pro-osteogenic (pro-angiogenic—HUVECS)		[51]
hsa_circ_0026827	DPSC*^4^	Human	**↑**	Differentiation/mineralization	Pro-osteogenic and pro-mineralization	x	[52]
circ_0062582	BMSC	Human	**↑**	Differentiation/mineralization/proliferation	Pro-osteogenic, pro-mineralization and pro-proliferation		[53, 54]
hsa_circ_0070562	BMSC^b^	Human		Differentiation/mineralization	Pro-osteogenic and pro-mineralization		[55]
hsa_circ_0001493	BMSC^b^	Human		Differentiation/mineralization	Pro-osteogenic and pro-mineralization		[55]
hsa_circ_0074834	BMSC^c^	Human	**↑**	Differentiation/mineralization (supernatant affect angiogenesis)	Pro-osteogenic and pro-mineralization (pro-invasion, pro-migration and pro-angiogenesis—HUVECS)	x	[46•]
circRUNX2/hsa_circ_0076694	BMSC	Human	**↑**	Differentiation/mineralization	Pro-osteogenic and pro-mineralization		[56]
circ_AFF4	BMSC	Human	**↑**	Differentiation/mineralization	Pro-osteogenic and pro-mineralization	x	[57]
circINO80	ASC	Human		Differentiation/mineralization	Pro-osteogenic and pro-mineralization		[58]
circ-ITCH	BMSC	Human	**↑**	Differentiation/mineralization	Pro-osteogenic and pro-mineralization	x	[42]
circRFWD2	ASC	Human		Differentiation/mineralization	Pro-osteogenic and pro-mineralization		[58]
hsa_circ_0006393	BMSC	Human		Differentiation	Pro-osteogenic	x	[59]
circRNA_0016624	BMSC	Human	**↑**	Differentiation/mineralization	Pro-osteogenic and pro-mineralization		[60]
circRNA_0048211	BMSC^d^	Human	**↑**	Differentiation/mineralization	Pro-osteogenic and pro-mineralization		[61]
circRNA_33287	MSMSCs*^5^	Human	**↑**	Differentiation/mineralization	Pro-osteogenic and pro-mineralization	x	[29]
circRNA124534	DPSC	Human	**↑**	Differentiation/mineralization	Pro-osteogenic and pro-mineralization	x	[62]
circSIPA1L1	DPSC	Human	**↑**	Differentiation/mineralization	Pro-osteogenic and pro-mineralization	x	[63]
circSIPA1L1	SCAP*^6^	Human	**↑**	Differentiation/mineralization	Pro-osteogenic and pro-mineralization		[64]
circVANGL1	BMSC	Human	**↑**	Differentiation	Pro-osteogenic		[65]
hsa_circ_0001421	ASC	Human	**↑**	Differentiation/mineralization	Pro-osteogenic and pro-mineralization		[66]
hsa_circ_0076906	BMSC	Human	**↑**	Differentiation/mineralization	Pro-osteogenic and pro-mineralization		[67]
hsa_circ_0076690	BMSC	Human	**↑**	Differentiation	Pro-osteogenic		[68]
hsa_circ_0001485	FOB 1.19	Human	**↑**	Differentiation/mineralization	Pro-osteogenic and pro-mineralization	x	[69]
circStag1/hsa_circ_0003626	BMSC^d^	Human/rat	**↑**	Differentiation/mineralization	Pro-osteogenic and pro-mineralization	x	[45]
circ_0024097	BMSC/MC3T3	- /mice	**↑**	Differentiation	Pro-osteogenic		[71]
circRNA-23525	ASC	Mice	**↑**	Differentiation/mineralization	Pro-osteogenic and pro-mineralization		[72]
circ-SLC8A1	BMSC	Mice	**↑**	Differentiation/mineralization	Pro-osteogenic and pro-mineralization		[73]
mm9_circ_009056	MC3T3	Mice	**↑**	Differentiation/mineralization/proliferation	Pro-osteogenic, pro-mineralization and anti-proliferation		[74]
mmu_circ_003795	MC3T3 and MDPC23	Mice	**↑**	Differentiation/mineralization	Pro-osteogenic and pro-mineralization		[75]
circ_0000020	BMSC	Rat	**↑**	Differentiation/mineralization/apoptosis	Pro-osteogenic, pro-mineralization and anti-apoptotic		[76]
circRNA-vgll3/circRNA-0879	ASC	Rat	**↑**	Differentiation/mineralization	Pro-osteogenic and pro-mineralization	x	[77]
circARSB	BMSC	Rat		Differentiation/mineralization	Pro-osteogenic and pro-mineralization		[78]
circFgfr2	DFC*^7^	Rat	**↑**	Differentiation/mineralization	Pro-osteogenic and pro-mineralization		[26]
circPTEN	BMSC	Rat		Differentiation/mineralization	Pro-osteogenic and pro-mineralization		[78]

Source: *^1^bone-marrow stem cells; *^2^adipose-derived stem cells; *^3^periodontal ligament stem cells; *^4^dental pulp stem cells; *^5^maxillary sinus membrane stem cells; *^6^apical papilla stem cells; *^7^dental follicle cells

Disease: ^a^from patients with osteonecrosis of the femoral head; ^b^from patients with ankylosing spondylitis and healthy donors; ^c^from patients with non-union; ^d^from patients with post-menopausal osteoporosis

## circRNAs that Inhibit Osteogenic Differentiation

During osteoblast differentiation of human bone marrow MSCs (BMSCs), Zhang et al. identified 3938 and 1505 circRNAs as upregulated and downregulated, respectively, when compared with BMSCs at day 0 [27]. Approximately 95% of these circRNAs were derived from protein coding genes, which functional annotation analysis was reported to be enriched in osteogenesis-associated terms and pathways, such as focal adhesion, ECM-receptor interactions, and calcium, insulin, and mTOR signaling pathways [27]. The authors also  reported that circIGSF11 (hsa-circRNA13685) is significantly decreased during osteogenesis, and its inhibition by siRNA positively affected osteogenic differentiation and the mineralization capacity [27]. In adipose-derived stem cells (ASCs), Huang et al. also investigated the circRNAs expression profile under osteogenic differentiation [28]. From a total of 210 differentially expressed circRNAs, 150 were upregulated, while 60 were downregulated [28]. Based on the* p* value, fold-change, and raw intensity, 6 circRNAs were selected and further validated, including circUSP53, circZBTB16, circXLOC_007414, circTIPARP (upregulated), circMCM3AP, and circPOMT1 (downregulated). Loss of function assays to evaluate the role of circPOMT1 and circMCM3AP showed that COLI and RUNX2 levels were enhanced, as well as ALP and deposition of calcium nodules, indicating that both circRNAs act as negative regulators of osteogenic differentiation [28].

While investigating the effect of melatonin (an hormone that regulates the circadian rhythm and bone metabolic processes [30]) on human BMSCs osteogenic differentiation, Wang et al. conducted RNA sequencing analysis, following treatment of human BMSCs with melatonin [31]. Among the 209 circRNAs significantly impacted by the treatment, circ_0003865 showed the most prominent decrease. Silencing of circ_0003865 induced the formation of calcium nodules in human BMSC in osteogenic-inducing conditions [31]. Also, it increased RUNX2, ALP, and OPN levels in in vitro assays and in a postmenopausal osteoporosis (PMOP) mouse model induced by ovariectomy (OVX). [31]. In agreement +, bone density was enhanced, compared to the control OVX mice, suggesting that circ_0003865 silencing restrains OVX-induced osteoporosis through modulation of the BMSC osteogenic potential [31].

Recently, it has been reported that in osteoporosis, MSCs not only show an impairment in their ability to differentiate into osteoblasts but also an enhancement in their adipogenic potential [32, 33]. In this context, silencing of hsa_circ_0006859 can be a promising therapeutic approach to promote bone formation due to the opposing roles on osteogenesis and adipogenesis [34••].

Besides their anti-osteogenic role, circHGF (hsa_circ_0080914) [35] and circUSP45 (hsa_circ_0077425) [36] were also described as suppressors of the proliferative capacity of hBMSC, whereas the circCDK8 (hsa_circ_0003489) was reported to additionally induce autophagy and apoptosis of periodontal ligament stem cells (PDLSC) in hypoxic conditions [37]. Interestingly, circUSP45 was described to be upregulated in human bone samples from patients with glucocorticoid-induced osteonecrosis of the femoral head (GIONFH) [36].

Although not in human primary cells, but in the MC3T3 cell line, circ_0058792 levels were shown to be significantly downregulated when cultured under osteogenic-inducing conditions. Silencing of circ_0058792 led to enhancement of the expression of Alp, Ocn, and Runx2 [38].

## circRNAs that Promote Osteogenic Differentiation

circRNA CDR1as (hsa_circ_0001946), one of the first circRNA described to have a miRNA sponge activity, was shown to be upregulated in human PDLSCs after osteogenic-induction, and to promote osteoblasts differentiation in vitro by reducing the expression of RUNX2, ALP, and alizarin staining via the miR-7/GDF5/SMAD and p38 MAPK signaling pathway [39]. In line with these results, siRNA-CDR1as treated PDLSCs, loaded on scaffolds and implanted in nude mice calvarial defects, resulted in decreased bone volume/trabecular volume (BV/TV) and bone mineral density (BMD), less bone formation in the defect area, and a smaller area stained positive for OCN [39]. Nevertheless, in another independent study by Chen et al., circRNA CDR1as was described as anti-osteogenic, since knockdown of CDR1as led to the upregulation of osteogenic markers (RUNX2, OSTERIX, BMP2, ALP, and OCN) and enhanced mineralization [40]. Additionally, CircRNA CDR1as was upregulated in BMSCs from patients with steroid-induced osteonecrosis of the femoral head (SONFH) and described to induce adipogenesis by affecting Adipsin, PPARγ, CEBPα, and FABP4 and oil red O staining, via the miR-7-5p/WNT5B pathway [40]. Conflicting results may be due to differences in the MSCs origin (bone-marrow or periodontal ligament) and healthy versus disease environment.

The circ-ITCH, previously reported to be upregulated during osteogenic differentiation of human PDLSCs [41], was described to have the same behavior in BMSCs, and to be downregulated in the bone marrow of osteoporotic patients [42]. In vitro experiments show that the ALP activity, the formation of mineralized nodules, and the mRNA and protein expression of osteogenic markers (RUNX2, OPN, and OCN) were enhanced in circ-ITCH overexpressing BMSCs [42]. Moreover, circ-ITCH controls osteogenesis by acting as miR-214 sponge, since miR-214 silencing reversed the effects of circ-ITCH knockdown [42]. In vivo experiments, testing the overexpression of circ-ITCH in OVX mice, led to increased BMD and enhanced mRNA expression levels of RUNX2, OPN, OCN, and YAP1, but decreased the expression of the anti-osteogenic miR-214 [43, 44], suggesting that circ-ITCH can ameliorate PMOP [42].

In clinical bone samples from patients with osteoporosis, the expression levels of circStag1 (hsa_circ_0003626) are impaired and positively correlate with BMD (T-score) and osteogenic markers (*ALP*, *OCN*, and *OPN*) [45]. Overexpression of this circRNA significantly promoted osteogenic differentiation (ALP staining and expression of osteogenic markers) and mineralization, while showing the contrary effect after circStag1 silencing in BMSCs, confirming its positive effect on osteogenesis [45]. Moreover, circStag1 interacts with the human antigen R (HuR), promoting its translocation into the cytoplasm, leading to the activation of the Wnt signaling pathway and enhancement of the osteogenesis, through stabilization of the low-density lipoprotein receptor-related protein 5/6 (Lrp5/6) and β-catenin expression [45]. Treatment of OVX rats with circStag1 resulted in increased levels of circStag1 in osteoporotic bone tissues, significantly decreased in OVX animals, and rescued the BMD, the number and density of osteoblasts, the trabecular bone area, and the cortical thickness, while preventing the reduction of new bone formation. Also, circStag1 restored the mineral apposition and bone formation rate, improved the bone mechanical properties (such as max force, stiffness, and max strength), and reestablished the levels of ALP, OCN, OPN, LRP5, LRP6, and β-catenin, while reducing the number of osteoclasts and serum levels of the bone resorption marker CTX-I. These results suggest that the treatment with circStag1 could prevent bone loss induced in OVX rats [45].

Ouyang et al. identified hsa_circ_0074834 as highly expressed in BMSCs isolated from normal bone fracture healing patients, in comparison to bone nonunion patients. It was also increased during osteogenesis [46•], suggesting its involvement in bone formation. The hsa_circ_0074834-overexpressing-BMSCs enhanced ALP (staining and activity), augmented mineralization, and induced the expression of RUNX2, COL1A1, and OCN, whereas the opposite was observed in hsa_circ_0074834-knockdown-BMSCs [46•]. Bioinformatic analysis predicted miR-942-5p to bind to hsa_circ_0074834 and to target ZEB1 and VEGF [46•]. Also, the authors revealed that the supernatant of hsa_circ_0074834-overexpressing-BMSCs was enriched in VEGF and enhanced HUVECs migration, invasion, and angiogenesis, while the supernatant from hsa_circ_0074834-knockdown-BMSCs inhibited all these processes. The ability of this circRNA to have a pro-osteogenic and regenerative effect in vivo was tested in a femoral monocortical defect model, where it was observed an increased BV/TV and BMD in the animals treated with hsa_circ_0074834-overexpressing-BMSCs [46•]. Another study identified a second circRNA, the hsa_circ_0006215, which was decreased in BMSCs from osteoporotic patients, acted as a ceRNA for miR-942-5p and to facilitate osteogenic differentiation [47••]. Likewise, the supernatant from hsa_circ_0006215-overexpressing-BMSCs significantly promoted angiogenesis, and in vivo results are in line with the in vitro experiments [47••].

Likewise, in human primary MSCs, several other circRNAs were reported as pro-osteogenic, such as circFOXP1 (hsa_circ_0001320) [48], circ_0001795 [49], circ_0005564 [25], circ_0011269 [50], circ_0019693 [51], hsa_circ_0026827 [52], circ_0062582 [53, 54], hsa_circ_0070562 [55], hsa_circ_0001493 [55], circRUNX2 (hsa_circ_0076694) [56], circ_AFF4 [57], circINO80 [58], circRFWD2 [58], hsa_circ_0006393 [59], circRNA_0016624 [60], circRNA_0048211 [61], circRNA_33287 [29], circRNA124534 [62], circSIPA1L1 [63, 64], circVANGL1 [65], hsa_circ_0001421 [66], hsa_circ_0076906 [67], and hsa_circ_0076690 [68]. Furthermore, circRUNX2 [56], hsa_circ_0006393 [59], and circFOXP1 (hsa_circ_0001320) [48] levels were shown to be impaired in bone from osteoporotic patients, whereas circ_0001795 [49] was described to be significantly reduced in the bone-marrow (Fig. 1, left panel). Several other functional studies with circRNA were performed in primary rat or mice MSC, or even cell lines [69–78]; nevertheless, validation in human MSC or osteoblasts is still needed.

Although the number of studies regarding the role of circRNAs on processes linked to bone formation increased substantially in the past few years, there is still a long way to go, when compared with the available information for other transcripts, such as miRNA.

## The Role of circRNAs as Regulators of Osteoclast Differentiation

Osteoporotic patients have a clear degradation of the bone components and structure [21, 79], mainly caused by the exacerbated activation of the osteoclasts, multinucleated cells from the hematopoietic myeloid lineage [80]. These cells are key in bone remodeling and the main responsible for bone resorption. [80]. Currently, the number of studies addressing the role of circRNAs in osteoclasts function is still limited. Future studies should focus on exploring the circRNA expression levels during osteoclastogenesis in human cohorts. Specifically, the identification of circRNA involvement in the different stages of monocytes to osteoclasts differentiation, as well as during the resorption process in human cells, could lead to the discovery of potential new targets for osteoporosis. So far, most of the studies have been performed in primary mouse osteoclasts or in the RAW264.7 cell line, providing the first insights into the role of circRNA in osteoclasts. Table 2 summarizes the current studies testing the role of circRNAs in osteoclast differentiation.

**Table 2:** Circular RNAs (circRNAs) involved in osteoclastogenic differentiation in in vitro studies

circRNA	Type of cells	Species	Expression during osteoclastogenic differentiation (**↑**/**↓**)	Biological process affected	Function/phenotype	In vivo	Ref
hsa_circ_0021739	PBMC*^1 a^	Human	**↓**	Differentiation	Anti-osteoclastogenic		[88]
circHmbox1/circ_0000549	BMM*^2^ and RAW264.7	Mice	**↓**	Differentiation (exosomes affect osteogenic differentiation)	Anti-osteoclastogenic	x	[85]
circBBS9/mmu_circ_0001757	BMM*^2^	Mice	**↑** (BMM mice) and human OC (compared to BMM)	Differentiation/resorption	Pro-osteoclastogenic and pro-resorption	x	[81••]
circ_0008542	RAW264.7	Mice		Differentiation/resorption	Pro-osteoclastogenic and pro-resorption	x	[86]
circRNA_009934	BMM	Mice	**↑**	Proliferation/differentiation	Pro-osteoclastogenic and pro-proliferation		[87]
circRNA_28313	BMM	Mice	**↑**	Differentiation	Pro-osteoclastogenic		[83]
circCHEK1_246aa	PBMC	Human		Differentiation	Pro-osteoclastogenic		[90]

Source: *^1^peripheral blood mononuclear cells; *^2^bone-marrow monocytes/macrophages

Disease: ^a^from healthy postmenopausal (PMOP) female patients

Wang et al. recently identified circBBS9 (mmu_circ_0001757), a highly conserved circRNAs, as having stage-specific functions in osteoclasts multinucleation. Following RNA sequencing, this circRNA was validated as being significantly increased in mononucleated preosteoclasts compared with bone marrow macrophages [81••]. The circBBS9 human homolog (hsa_circ_0134188) is also upregulated in osteoporotic human bone samples versus healthy individuals, as well as in peripheral blood mononuclear cells-derived osteoclasts [81••]. Stabilization of the expression at later stages of the differentiation process implied a crucial role during the multinucleation stage [81••]. The stage-specific function on the multinucleation process was confirmed by silencing circBBS9 at different stages of the differentiation process [81••]. Results showed that when circBBS9 was silenced at day 3 (mononucleated cells), the number of TRAP + preosteoclast and multinucleated osteoclast was impaired, as well as the percentage of resorbed area, in opposition to inhibition at day 5 (presence of multinucleated cells), where no obvious changes were observed [81••]. Also, the levels of key transcription factors (NFATc1 and c-FOS) and bone resorption–related proteins (integrin-D3, CTSK, and V-ATPase-d2) were repressed following circBBS9-knockdown. To evaluate these findings in vivo, OVX mice were intravenously injected with the engineered nanoparticles for circBBS9 silencing, designed as siRNA-circBBS9 loaded nanoparticles with mononucleated preosteoclast membrane encapsulation [81••]. The authors show that treatment with these particles prevented bone loss and osteoclast formation, resulting in a significant decrease of the bone surface covered with osteoclasts and the osteoclast number, independently of the treatment starting 1 or 5 weeks after the OVX [81••].

Dou et al. investigated the expression of several classes of transcripts, including circRNAs, using microarray screening, during the different stages of osteoclastogenesis in the murine monocytic RAW264.7 cell line [82]. From a total of 1797 circRNAs detected, 256 were differentially expressed in pre-osteoclasts, 213 in mature osteoclasts, and 156 in activated osteoclasts, compared with non-differentiated RAW264.7 cells [82]. When considering the analysis of differentially expressed circRNAs across all the osteoclastogenic differentiation stages, the authors identified 19 upregulated and 5 downregulated circRNAs [82]. These results suggest a stage-specific role for the majority of the circRNAs, while only a few are relevant in all stages of the osteoclasts differentiation.

Another independent study used microarray profiling to compare circRNA levels in primary murine differentiated and non-differentiated BMM and identified 29 upregulated and 52 significantly downregulated circRNAs [83]. Among the differentially expressed circRNAs, circRNA_012460, circRNA_28313, circRNA_28312, circRNA_28309, circRNA_001034, circRNA_21447, circRNA_40206, and circRNA_28236 were selected for further validation, being circRNA_28313 the most upregulated and the selected candidate for loss-of-function studies [83]. circRNA_28313 knockdown hindered the osteoclasts differentiation by impairing the number of TRAP^+^ multinuclear cells, the actin ring formation (essential for bone resorption), and the expression of CSF1, PU.1, TRAP, NFATc1, and CTSK, when compared with the control group [83]. The authors further discovered that circRNA_28313 acts as ceRNA for miR-195a, relieving miR-195a-mediated suppression of CSF1. Knockdown of circRNA_28313 in vivo, through tail vein injection, significantly suppressed OVX-induced bone resorption [83]. Micro-CT 3D reconstruction shows more trabecular bone and improved histomorphometric parameters (BV/TV, trabecular thickness, and trabecular number) in circRNA_28313-knockdown-OVX-mice. The number of TRAP-positive osteoclasts and bone loss was significantly reduced when circRNA_28313 levels were decreased in comparison with the OVX control group. Serum levels of RANKL and CSF1, two required cytokines for differentiating progenitor cells into osteoclasts [84], and TRAP activity, were significantly decreased in the circRNA_28313-knockdown-OVX mice compared with control. [83]. Therefore, both in vitro and in vivo results support the hypothesis that circRNA_28313 knockdown prevents OVX-induced bone loss.

Liu et al. also used an OVX mice model to evaluate the functional effect of circHmbox1 (circ_0000549), which is downregulated in mice osteoclastogenesis induced by TNF-α [85]. Specifically, in vitro experiments showed that circHmbox1 enhanced RANKL-induced primary osteoclast differentiation by increasing the expression of *TRAP*, *CTSK*, and *NFATc1*, while circHmbox1 overexpression had the contrary effect [85]. OVX mice injected with circHmbox1 showed improved BMD and BV/TV ratio, whereas the TRAP-stained area of the bone sections was decreased, compared with control animals [85]. Likewise, dynamic bone formation parameters, such as mineral apposition rate and mineralizing surface, were increased in the circHmbox1 group, compared with mock-treated OVX mice, suggesting that the overexpression of circHmbox1 in vivo not only alleviates OVX-induced osteoporosis, but also promotes bone formation [85]. Since bone formation and bone resorption are two processes that occur continuously and unceasingly throughout life, the authors tested the impact of circHmbox1 in intercellular communication. Specifically, an increased expression of *RUNX2* and *OSX* and ALP staining was found when osteoblasts were cultured with exosomes derived from circHmbox1-overexpressing RAW264.7 [85]. Wang et al. also investigated circRNA mediated communication via exosomes. In this study, exosomes derived from circ_0008542-overexpressing-MC3T3 (mouse pre-osteoclasts) lead to an increase in c-Fos, NFATC1, RANK, ACP5 (gene encoding TRAP), CTSK, and MMP9 levels, as well as a higher number of nuclei per osteoclast [86]. Administration of exosomes, derived from circ_0008542-overexpressing-MC3T3, in mice tail vein caused an increase in the number of osteoclasts and accentuated bone loss, 8 weeks after injection [86]. Further research pursuing the study of bidirectional osteoclasts-osteoblasts communication mediated by circRNAs is needed.

Other circRNAs have been tested in in vitro studies. Among those, circRNA_009934 inhibition impairs murine BMMs osteoclastogenesis, when stimulated with RANKL and M-CSF for 3 days, by changing mRNA and protein levels of differentiation-associated genes, including TRAF6 and c-FOS and bone resorption–related genes, such as CTSK and MMP9. [87]. Also, hsa_circ_0021739 showed decreased expression during osteoclastogenic differentiation of human peripheral blood mononuclear cells (PBMCs) and negatively impacted the number of osteoclasts [88]. Secondary osteoporosis often develops in multiple myeloma patients due to the exacerbated activity of the osteoclasts induced by the malignant plasma cells [89]. Gu et al. discovered that multiple myeloma cells expressed circCHEK1_246aa (a circRNAs with protein-coding capacity) and that its overexpression increases the number of TRAP-positive human PBMCs [90].

Overall, understanding the role of circRNA in osteoclasts fusion and resorption, particularly in human primary cells, should be further investigated for the future potential use of circRNA as therapeutic targets in osteoporosis.

## Circulating circRNAs as Biomarkers of Osteoporosis

The potential use of circRNAs as biomarkers for osteoporosis diagnosis or prognosis has been raising interest. circRNAs have been found to be deregulated in several human pathologies [91–94]. These transcripts can be detected in body fluids, using minimally invasive methods [95–97]. An advantage of circRNA as circulating biomarkers of diseases is the high stability of these molecules compared with linear RNA. In fact, the circular conformation provides an enhanced ribonuclease-resistance [98]. Currently, several studies explored the potential use of circulating circRNAs as biomarkers of human osteoporosis [34••, 50, 51, 60, 65, 67, 99], but those have been mainly limited to blood/plasma/serum samples, while the detection of circulating circRNAs in urine or saliva for the diagnosis of osteoporosis is yet to be pursued. Figure 1 (right panel) summarizes circulating circRNA in human osteoporotic samples that have been validated.

RNA-sequencing revealed that circulating circRNAs exhibit distinct patterns in osteoporotic patients compared with control donors [60, 68, 100]. Whole transcriptome sequencing of PBMC from old male patients (more than 60 years old) found 398 circRNAs to be differentially expressed, when compared to healthy age-matched controls [100]. In particular, hsa_circ_0042409 was validated to be highly expressed in osteoporosis patients [100]. Yu and Liu identified 211 upregulated and 176 downregulated circRNAs in serum/plasma of PMOP patients versus matched healthy controls [60]. Among these, circRNA_0016624 was validated as being decreased in osteoporosis and to act as a miR-98 sponge, enhancing the expression of the osteogenic gene BMP2 [60]. Also, Han et al. detected that hsa_circ_0076690 and hsa_circ_0111433 were markedly reduced in osteoporotic patients and were inversely correlated with BMD and *T*-score [68]. Nevertheless, only hsa_circ_0076690 show a potentialt diagnostic value [68]. Following microarray, circ_0011269 was reported to be diminished in the plasma/serum of osteoporotic patients [50]. Resorting only to male participants, Huang et al. identified a total of 237 circRNAs to be differentially expressed between osteoporotic and healthy patients in plasma/serum [101]. The aberrant upregulation detected for circ_0006873 and circ_0002060 by microarray was validated by PCR [101]. Although both circRNAs were negatively correlated with BMD and *T*-score, only circ_0002060 showed potential diagnostic value [101]. Also, Zhao et al. identified hsa_circ_0001275 in PBMCs as a biomarker of PMOP [8]. In silico analysis of PMOP-related datasets lead to the validation of hsa_circ_0023417, hsa_circ_0078309, hsa_circ_0063533, and hsa_circ_0036760 as differently expressed in human PBMCs between the PMOP and the control group [102].

In a targeted study, circVANGL1 was discovered to be downregulated in the serum of patients diagnosed with osteoporosis, by quantitative reverse-transcriptase PCR (qRT-PCR) [65]. Likewise, circ_0001445 was identified as decreased in plasma of patients suffering from osteopenia or osteoporosis. Further analysis shows that it could be used to distinguish between osteopenic or osteoporotic patients from the healthy controls [99]. Moreover, circ_0001445 was shown to be positively correlated with the *T*-score, whereas negatively correlated with the bone turnover marker β-isomerized C-terminal telopeptides (β-CTx). In addition, the expression levels of circ_0001445 increased in PMOP patients undergoing 6 months treatment with anti-osteoporotic drugs, indicating a potential role in the treatment evaluation [99].

Recently, exosomes emerged as biomarkers with clinical significance and several studies show that circRNA are enriched and stable in exosomes. Using a microarray, Zhi et al. measured the expression levels of circRNAs in exosomes isolated from the serum of both osteopenic and osteoporotic patients versus healthy controls [34••]. The study highlights that hsa_circ_0006859 was increased in exosomes from the osteopenic and osteoporotic patients. hsa_circ_0006859 has a negative correlation with bone density parameters. In addition, this circRNA may distinguish osteoporotic patients from patients with osteopenia and healthy controls, as well as be used to differentiate osteopenic patients from healthy controls, with high sensitivity and specificity [34••]. Additionally, the authors show that hsa_circ_0006859 levels were significantly decreased in the patients that received anti-osteoporotic treatment, compared with the levels at the beginning of the treatment [34••]. The anti-osteogenic and pro-adipogenic role of hsa_circ_0006859 [34••] highlights the potential that this circRNA holds for monitoring disease progression and as a therapeutic target.

In theory, circulating biomarkers should translate the changes at the bone tissue level. This is the case of hsa_circ_0076906 [67] and circ_0019693 [51] that are downregulated in both bone and serum samples from osteoporotic patients, compared with controls. Moreover, both have a pro-osteogenic function on human BMSC [51, 67].

For an exhaustive and robust future clinical use of circRNA as osteoporosis biomarkers, several parameters should be taken into consideration, including the biomarkers specificity and sensibility, the correlation with clinicopathological and histomorphometric variables, and the different treatments at the time of sample collection. Prognostic markers are also urgently needed. Additionally, the experimental conditions associated with collection and processing of samples from osteoporosis patients should be disclosed in detail, such as (1) the type of samples; (2) how the samples were obtained and the time until further processing; (3) the patients exclusion and inclusion criteria; and (4) the detailed description of the control group.

## Conclusions and Future Perspectives

Although there is a growing number of studies using high throughput tools, such as RNA sequencing and microarrays, to detect circRNAs in either osteoporotic patients or during the osteogenic and osteoclastogenic differentiation processes, there are several aspects that require further consideration: (1) to implement detailed and standardized guidelines for an uniform circRNA nomenclature, ensuring the proper annotation in databases and avoiding redundancies and/or misannotations; (2) to increase the number of human cohorts of osteoporosis patients pinpointing the potential use of circRNAs as biomarkers and to perform a more in-depth analysis demonstrating their latent significance as biomarkers; (3) to further examine the impact of circRNA in osteoclastogenic differentiation in human primary cells and, most importantly, on the bone resorption process; and (4) to perform wide screen analysis of the molecular targets affected by the modulation of circRNAs (e.g., proteomic analysis) to clarify their main regulatory pathways and mechanisms.
